# Strain-level resolution and pneumococcal carriage dynamics by single-molecule real-time (SMRT) sequencing of the *ply*NCR marker: a longitudinal study in Swiss infants

**DOI:** 10.1186/s40168-022-01344-6

**Published:** 2022-09-22

**Authors:** Oluwaseun Rume-Abiola Oyewole, Philipp Latzin, Silvio D. Brugger, Markus Hilty

**Affiliations:** 1 Institute for Infectious Diseases, University of Bern, Friedbühlstrasse 51, 3001 Bern, Switzerland; 2 Graduate School for Cellular and Biomedical Sciences, University of Bern, Bern, Switzerland; 3 Division of Respiratory Medicine, Department of Pediatrics, Inselspital, University of Bern, Bern, Switzerland; 4 Department of Infectious Diseases and Hospital Epidemiology, University Hospital Zurich, University of Zurich, Zurich, Switzerland

**Keywords:** Respiratory microbiome, PacBio single-molecule real-time (SMRT) sequencing, DADA2, Co-colonization, *Streptococcus pneumoniae*, Carriage duration, First year of life

## Abstract

**Background:**

Pneumococcal carriage has often been studied from a serotype perspective; however, little is known about the strain-specific carriage and inter-strain interactions. Here, we examined the strain-level carriage and co-colonization dynamics of *Streptococcus pneumoniae* in a Swiss birth cohort by PacBio single-molecule real-time (SMRT) sequencing of the *ply*NCR marker.

**Methods:**

A total of 872 nasal swab (NS) samples were included from 47 healthy infants during the first year of life. Pneumococcal carriage was determined based on the quantitative real-time polymerase chain reaction (qPCR) targeting the *lytA* gene. The *ply*NCR marker was amplified from 214 samples having *lytA*-based carriage for pneumococcal strain resolution. Amplicons were sequenced using SMRT technology, and sequences were analyzed with the DADA2 pipeline. In addition, pneumococcal serotypes were determined using conventional, multiplex PCR (cPCR).

**Results:**

PCR-based *ply*NCR amplification demonstrated a 94.2% sensitivity and 100% specificity for *Streptococcus pneumoniae* if compared to *lytA* qPCR. The overall carriage prevalence was 63.8%, and pneumococcal co-colonization (≥ 2 *ply*NCR amplicon sequence variants (ASVs)) was detected in 38/213 (17.8%) sequenced samples with the relative proportion of the least abundant strain(s) ranging from 1.1 to 48.8% (median, 17.2%; IQR, 5.8–33.4%). The median age to first acquisition was 147 days, and having ≥ 2 siblings increased the risk of acquisition.

**Conclusion:**

The *ply*NCR amplicon sequencing is species-specific and enables pneumococcal strain resolution. We therefore recommend its application for longitudinal strain-level carriage studies of *Streptococcus pneumoniae*.

Video Abstract

**Supplementary Information:**

The online version contains supplementary material available at 10.1186/s40168-022-01344-6.

## Introduction

Carriage studies are essential for monitoring the sero- and molecular type changes of circulating pneumococcal strains, especially in the current era of pneumococcal conjugate vaccines (PCVs). However, reliance on conventional, culture-based methods with a known bias towards dominant strains [1] may limit the perception of the true and full dynamics (multiple carriage, new acquisition versus unmasking, etc.) of pneumococcal strains in the nasopharynx. Although the development of more sensitive measures like the microarray has improved serotype detection [2], strain-level insights are still necessary for pneumococcal carriage studies, as they can aid in identifying and tracking strains across samples (independent of the serotype) and easily distinguish new strain acquisitions from old strain re-acquisitions, identifying potential sources of transmission.

Previous studies have attempted to ascertain pneumococcal profiles from nasopharyngeal samples via shotgun metagenomics [3, 4]; however, resolution of “unique” pneumococcal strains remained limited, as shotgun metagenomics requires a very high read coverage to detect minor variants, which is even more challenging to achieve with samples that have a high quantity of host DNA. Alternative approaches are therefore needed for a thorough exploration of *S. pneumoniae* strains and their dynamics during carriage.

In recent years, amplicon-based high-throughput sequencing (HTS) via Illumina, PacBio, etc. has revolutionized a plethora of culture-independent gut-microbiome investigations, offering insights on strain-level diversity and resolving structures of complex, microbial communities [5, 6]. This increased resolution (beyond the 16S rRNA level) is assured by using a species-specific marker gene as a measure of inter-strain genomic variability. Strains are then subsequently identified based on the presence or absence of at least one single nucleotide variant (SNV) via computational methods such as the Divisive Amplicon Denoising Algorithm (DADA2) [7, 8], oligotyping [9, 10], and UNOISE [11]. Although proven successful in microbiome studies, this strategy is yet to be applied to longitudinal carriage studies seeking to explore finer-grained dynamics of *S. pneumoniae* in the nasopharynx.

The ~ 1300-bp pneumolysin non-coding region (*ply*NCR) has been previously reported as a highly specific and conserved marker for *S. pneumoniae* [12–14]. The resolution of the *ply*NCR region for strain typing was found to be comparable with multi-locus sequence typing (MLST) [12]. In addition, the *ply*NCR region has also been investigated and validated for the detection of co-colonization using terminal restriction length polymorphism (T-RFLP) [13]. However, the usefulness of the *ply*NCR has so far only been tested by restriction enzymes, which may limit the strain resolution, as polymorphisms of specific enzyme cuts are usually low [13, 15].

In this study, we aimed to assess the application of PacBio single-molecule real-time (SMRT) sequencing of the *ply*NCR marker for pneumococcal strain-level resolution. Using nasal swab (NS) samples of infants in a longitudinal cohort, we reveal an accurate and in-depth visualization of strain-resolved pneumococcal profiles and associated serotypes during carriage over a 12-month period, including estimations for acquisition and carriage duration since the introduction of the 13-valent pneumococcal conjugate vaccine (PCV13) in Switzerland.

## Materials and methods

### In silico screening of the *ply*NCR marker in pneumococcal and streptococcal genomes

Using a perl script (https://github.com/egonozer/in_silico_pcr), an in-silico search for the *ply*NCR marker was performed on 8325 reference sequences (RefSeq) of *S. pneumoniae* genomes available on the public NCBI database (https://www.ncbi.nlm.nih.gov/genome/?term=Streptococcus+pneumoniae, accessed on March 25, 2021). For a specificity assessment, the *ply*NCR marker was also screened against the Genbank assembly database (https://www.ncbi.nlm.nih.gov/assembly) of the viridans group streptococci (VGS): *S. mitis* (138), *S. pseudopneumoniae* (87), *S. infantis* (12) and *S. oralis* (122). Extracted sequences with indel regions were verified by mapping published NCBI assemblies to raw reads available on the sequence read archive (SRA) using minimap2 v2.17 and visualizing the mapped regions corresponding to the *ply*NCR on the Integrative Genomics Viewer (IGV). Sequences with unverifiable indel regions due to an absence of data on the sequence read archive (SRA) or low read coverage mapping (< 10 reads per site) were excluded. Final sequences were aligned using muscle v3.8.1551 [16], and a maximum likelihood (ML) phylogenetic tree was constructed under the generalized time-reversible (GTR) + gamma model using fasttree v2.1.10 [17]. Tree annotation and visualization were performed using the interactive tree of life (iTOL) [18].

### Study participants, swab collection, and pneumococci screening

A total of 48 infants, who participated in our previous study [19] and were enrolled in the Basel Bern Infant Lung Development (BILD) Cohort (www.bild-cohort.ch) were followed during the first year of life [20]. Starting with the fifth week after birth [initiation time point; (*T*_0_)], nasal swabs (Verridial E. Mueller, Blonay, Switzerland) were collected biweekly from the study infants between February 2010 and April 2014 as previously described [19]. Ethical approval was obtained from the Ethics Committee of the Canton of Bern.

DNA was extracted directly from swabs (QIAamp DNA Minikit, Qiagen, Hilden, Germany) in 200 μl of transport medium and screened for pneumococci via quantitative real-time PCR (qPCR) targeting the *lytA* [21, 22]. A negative control was included for each qPCR run. The lower limit of detection (LLD) was defined as 10 *lytA* copies, and samples having < 10 *lytA* copies in 2/3 triplicate measurements were presumptively ruled as being negative for *S. pneumoniae*.

### Mock control design

**Fig. 1:**
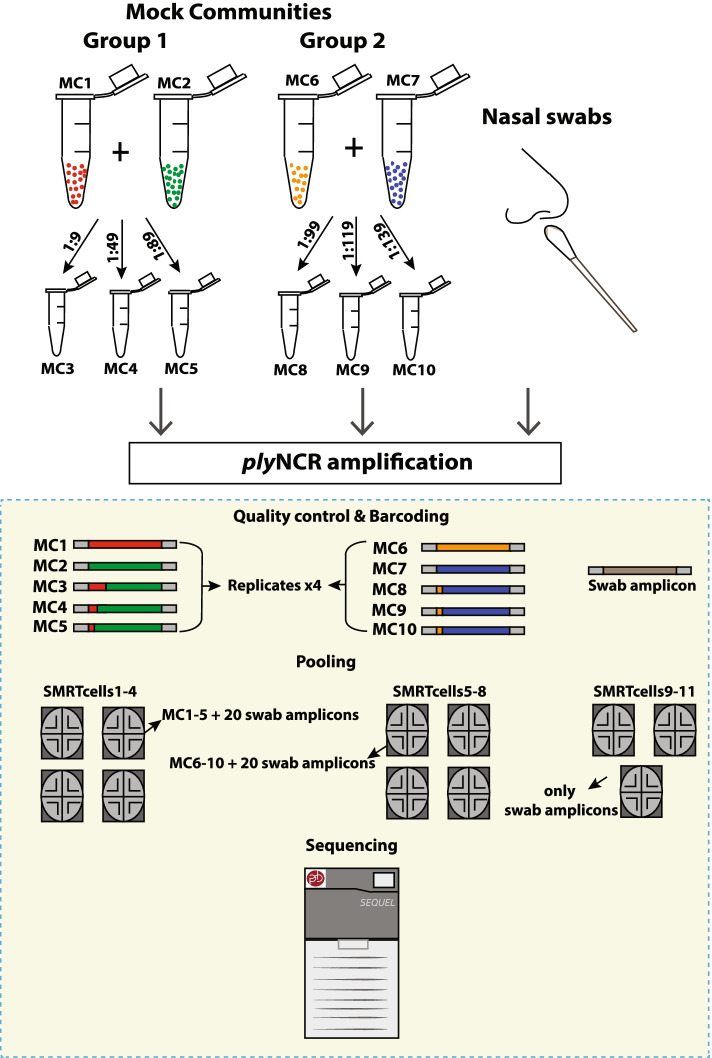
Experimental design of mock controls for *ply*NCR sensitivity. Ten mock communities were created using in-house pneumococcal strains of known composition and genomic signatures. The samples were prepared for *ply*NCR amplicon sequencing and sequenced in quadruplicates as controls together with nasal swab amplicons on the PacBio Sequel

Four non-encapsulated *S. pneumoniae* strains of known *ply*NCR genomic signatures were chosen from our previous study [23] (Additional file 1: Table S1). Genomic DNA of strains were diluted to equal masses (1 ng) and mixed in varying proportions to ascertain the resolution limit and sensitivity of the *ply*NCR as an amplicon marker during SMRT sequencing (Fig. 1). Mock communities (MC) were prepared as follows: (group 1) MC1–5—MC1 and 2; single strains and MC3, 4, and 5; mixtures of MC1 and 2 in ratios 1:9, 1:49, and 1:89, respectively; (group 2) MC6–10—MC6 and 7; single strains and MC8, 9, and 10; mixtures of MC6 and MC7 in ratios 1:99, 1:119, and 1:139, respectively.

### PCR for *ply*NCR marker amplification

PCR amplification of the *ply*NCR marker was performed using previously described primers [12], modified with tailed PacBio Universal sequences for downstream amplicon sequencing (Table 1). The *ply*NCR PCR reaction and cycling conditions were followed for in vitro samples (mock communities) and NS swabs as previously described [13], with the only modification being that 10 μl of swab DNA was used per reaction and 40 amplification cycles were used for NS samples with low yields from the first amplification.

**Table 1:** Primers used in this study

Gene	Primer	Sequence (5′-3′)	Reference
*lytA*	Forward	ACGCAATCTAGCAGATGAAGCA	[22]
	Reverse	TCGTGCGTTTTAATTCCAGCT	
	Probe	FAM-GCCGAAAACGCTTGATACAGGGAG-BHQ1	
*ply*NCR^a^	Forward	[GCAGTCGAACATGTAGCTGACTCAGGTCAC]AAAGGCTGCACGGACATTG	[12]
	Reverse	[TGGATCACTTGTGCAAGCATCACATCGTAG]CCGATTTGCCACTAGTGCGTAAGC	
*cpsA*	Forward	GCAGTACAGCAGTTTGTTGGACTGACC	[24]
	Reverse	GAATATTTTCATTATCAGTCCCAGTC	

^a^Pacbio Universal Sequence overhang in []. Primers were HPLC purified and blocked at the 5′ site with an amino group

PCR products were visualized on a 1% agarose gel and samples having the expected *ply*NCR amplicon product size of ~ 1.3 kb were purified using the QIAcube (Qiagen, Germany) or AMPure PB Beads (Beckman Coulter Inc., USA) and quantified on the Agilent 2100 Bioanalyzer (Agilent Technologies, Palo Alto, CA).

### PacBio SMRT amplicon sequencing of the *ply*NCR marker

Equimolar pooling of amplified DNA was performed using the Barcoded Universal F/R Primers Plate 96 v2 (Pacific Biosciences, USA) to allow multiplexing in pools of 25–26 amplicons per SMRTcell (Additional file 1: Table S2). In total, 11 SMRTcells were used. Four technical replicates of each mock control group (MC1–5 and MC6–10) were included in four respective SMRTcells per group (Fig. 1; Additional file 1: Table S2).

SMRTbell libraries from the pooled amplicons were prepared using SMRTbell Express Template Prep Kit 2.0 according to the manufacturer’s instructions (Pacific Biosciences, USA). Sequencing on the Sequel I platform was performed using the Sequel Binding kit 3.0, Sequel Sequencing Kit 3.0, and Sequencing Chemistry S/P3-C3/5.0. Each pool of purified SMRTbell libraries was sequenced on single 10-h movie SMRT cells (SMRT cell 1M v3) with a pre-extension time of 1.3 h.

### Bioinformatics and sequence analysis

Subreads from each SMRTcell were first demultiplexed with Lima v1.10.0 (https://github.com/PacificBiosciences/barcoding) and then aligned to an extracted *ply*NCR sequence region from the reference strain 106.66 (NCBI BioProject Accession: PRJNA554545) using Pbalign v0.4.1 (https://github.com/PacificBiosciences/pbalign/) with the parameters *hitPolicy*=allbest and *minAnchorSize*=19. Circular consensus sequence (CCS) reads were generated from the aligned subreads with *minPredictedAccuracy* = 0.999 using the CCS v4.0.0 (https://github.com/PacificBiosciences/ccs). All bioinformatic analyses were conducted on UBELIX (http://www.id.unibe.ch/hpc), the high-performance computing (HPC) cluster at the University of Bern. All raw PacBio sequencing reads were deposited in the European Nucleotide Archive (ENA) under study accession number PRJEB45241.

The R package, DADA2, was used to denoise the CCS reads and identify the relative abundance of each amplicon sequence variant (ASV) following the online DADA2 + PacBio protocol by Callahan (https://benjjneb.github.io/LRASManuscript/LRASms_Zymo.html). Data was analyzed per SMRTcell. ASVs were further processed by length exclusion (≤ 1150 bp) and false-positive removal (Additional file 1: Fig. S1). For the finalized ASV output, ASVs sharing 100% identity were defined as “unique.”

### Molecular serotype/serogroup identification

Serotypes/serogroups were determined via conventional PCR (cPCR) either in singleplex or multiplex for all samples having ≥ 10 *lytA* copies, based on established protocols by the Streptococcus Laboratory of the Centers for Disease Control and Prevention (CDC; https://www.cdc.gov/streplab/pneumococcus/resources.html). Samples with ≥ 10 *lytA* copies but no serotype and no *cpsA* amplification bands were considered non-typeable pneumococci (NT). We found no samples with missing serotype but presenting *cpsA* bands. In samples, where co-colonization was present (≥ 2 *ply*NCR ASVs) but only one serotype was identified by cPCR, the identified serotype was assumed to belong to the most abundant ASV and the presumed unidentified serotype(s) were classified as “unknown.” The term “unknown” was used due to the possibility of the presumed unidentified serotype(s) being (i) the same serotype as the most abundant ASV, (ii) a NT, or (iii) a serotype not included in the 40-serotype multiplex primer panel.

### Data analyses

Pneumococcal carriage prevalence was defined as the proportion of infants with ≥ 10 *lytA* copies in a swab sample. Time to acquisition, carriage duration, and clearance were calculated based on previous definitions [25, 26]. In brief, the time of acquisition was estimated to be the midpoint from the last of two negative swabs to the first positive swab while clearance was inferred as the midpoint between the last positive swab and the first of two negative swabs. Time of acquisition for infants with detectable pneumococci at *T*_0_ (first swab taken) was inferred to be the midpoint from birth to *T*_0_. The period between acquisition and clearance was considered the duration of carriage, and this was calculated for both pneumococcal strains (unique *ply*NCR ASV) and serotypes.

Statistical analyses were conducted in the R studio environment v3.6.1 (https://www.r-project.org/). Time to first acquisition and carriage duration were analyzed using the Kaplan-Meier survival curve. The Cox regression model was used to assess the association between time to first acquisition and epidemiologic/socio-economic factors such as respiratory symptoms, smoking exposure, season of birth, parental education, having two or more siblings, and pneumococcal vaccination. Carriage duration was right censored at the last swab time point. New acquisitions at the final swab time point were excluded from the duration analyses. Co-colonization dynamics were categorized and adapted from previous definitions [27]: (i) strain displacement (a primary colonizer is outcompeted by the secondary strain and subsequently, cleared), (ii) strain dominance (carriage of a primary colonizer is maintained and newly acquired secondary strains are transient), (iii) stable co-colonization (two strains are carried across ≥ 2 time points), and (iv) short-term co-colonization (multiple strains are observed at a single time point). The chi-squared test was applied for categorical testing and *P* < 0.05 was considered significant. The results were reported with 95% confidence intervals (CI).

## Results

### In silico evaluation of the *ply*NCR marker reveals pneumococcal specificity and *ply*NCR homolog presence among viridans group streptococci (VGS)

**Fig. 2:**
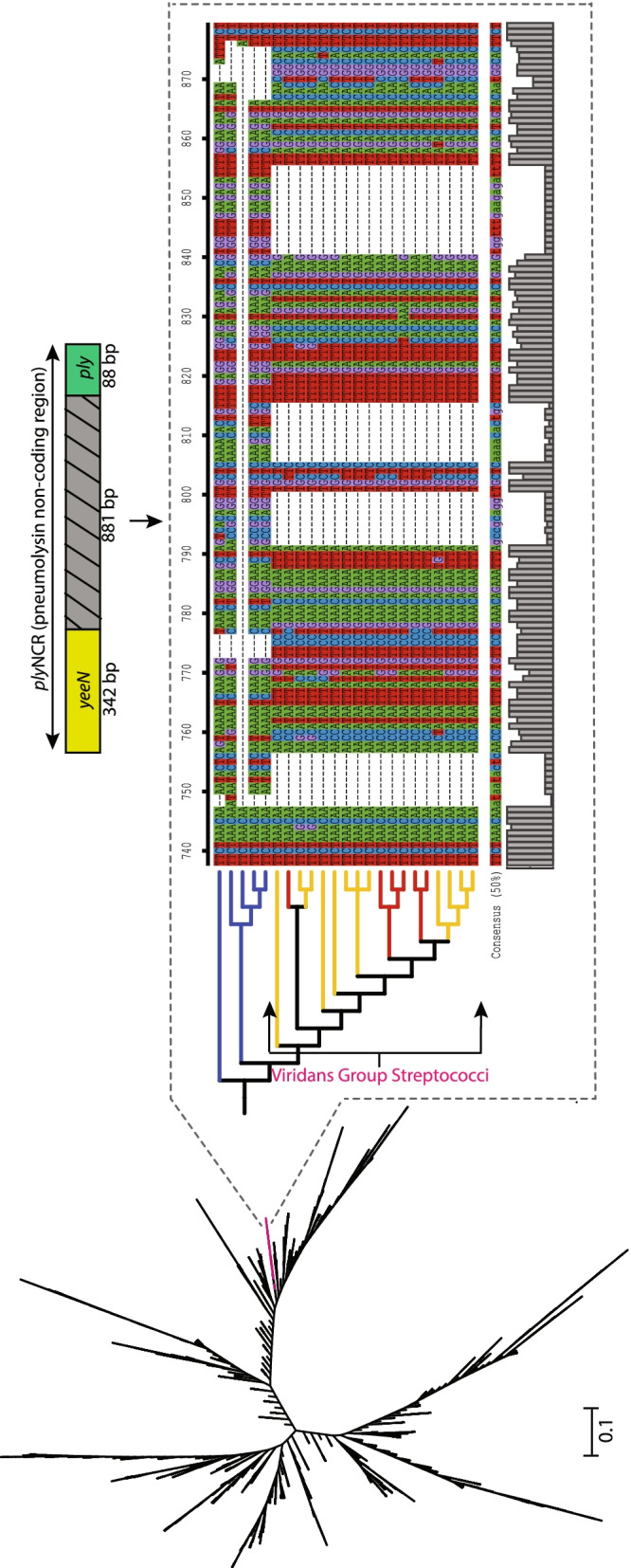
Maximum likelihood phylogeny of the *ply*NCR marker among strains of the *Streptococcus mitis* group. The ML tree was inferred using the generalized time reversible (GTR) + gamma model with FastTree and is unrooted with unscaled branches. A zoomed-in subtree (dashed rectangle) shows a cladistic clustering and a portion of the sequence alignment from five pneumococci (in blue) and 18 viridans group streptococci (VGS; *S. mitis* and *S. pseudopneumoniae* in red and yellow, respectively) with the latter having characteristic deletions. Above the subtree, the *ply*NCR is illustrated as a non-coding region flanked by partial sections of the conserved genes, *yeeN* (hypothetical regulatory protein) and *ply* (pneumolysin)

In order to investigate the presence and exclusivity of the *ply*NCR marker to pneumococcal genomes, an in silico screening of the marker was performed on all *S. pneumoniae* RefSeqs as well as Genbank assemblies of the closely related VGS species. Of the 8325 pneumococcal genome RefSeqs on the NCBI database (accessed, March 25, 2021), the *ply*NCR marker was present in all but one genome (0.01%; accession: GCF_001113845.1), which only gave a partial hit (~ 500 bp), due to the presence of multiple large deletions, which is likely indicative of inadequate sequencing coverage prior to assembly. Among non-pneumococcal streptococci, no hits for the marker were found against *S. oralis* and *S. infantis* genomes. However, homologous of the marker, which were identical in size (~ 1100 bp), were present in 6/138 (4.3%) *S. mitis* and 12/87 (13.8%) *S. pseudopneumoniae* genomes. All homolog sequences clustered separately due to similar deletions and were within the same clade as five pneumococcal *ply*NCR sequences, which also exhibited deletions (Fig. 2; Additional file 1: Table S3). The deletions in these five pneumococcal *ply*NCR sequences were not identical to the homologs but were confirmed by mapping the raw reads to the assemblies. Therefore, the in silico specificity for the “right size” *ply*NCR is 100% for *S. pneumoniae*.

### Comparison of *ply*NCR PCR to the *lytA* qPCR for pneumococcal detection in NS samples

Given a demonstrated in silico specificity of the *ply*NCR among *S. pneumoniae* genomes, we next compared the PCR detection of the *ply*NCR to the qPCR-based assay targeting the highly conserved *lytA* gene using NS samples from 47 BILD cohort infants (samples from one infant were excluded due to low quality as shown below). Between February 2010 and April 2014, a total of 1068 NS were collected. After exclusion of low-quality samples and those taken during antibiotic therapy or respiratory infection (*n* = 196), a total of 872 high-quality NS samples were included from 47 infants with a median of 19 samples per infant (range, 11–25). The full characteristics of the 47 BILD cohort infants are presented in Additional file 1: Table S4. Overall, 631/871 (72.4%) high-quality NS samples had < 10 *lytA* copies and were considered negative for pneumococci. This was corroborated by negative *ply*NCR (617/631 samples) or “wrong size” (~ 1100 bp; Additional file 1: Fig. S2) (14/631 samples) amplification in all samples, indicating a 100% specificity of the marker PCR. Of the remaining 240 (27.6%) samples with ≥ 10 *lytA* copies, only 14 (5.8%) failed to amplify the *ply*NCR marker by PCR, resulting in a sensitivity of 94.2% (Additional file 1: Table S5).

### PacBio amplicon sequencing for strain (*ply*NCR) resolution

**Fig. 3:**
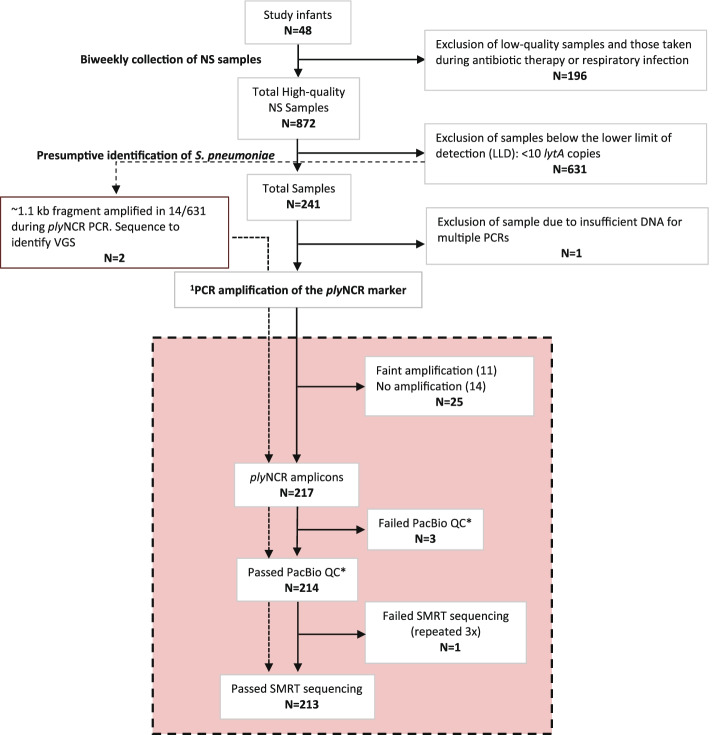
Flowchart of nasal swab (NS) sample processing for PacBio SMRT sequencing. Molecular processing of NS samples for *ply*NCR sequencing (strain identification) highlighted in peach. ^1^PCR performed using primers with PacBio universal sequence overhang. *QC: quality control excludes amplicons with no observable peak or multiple peaks on the fragment analyzer. VGS, viridans group streptococci

After exclusion of low-yield amplicons (i.e., those that produced faint bands on the gel) and low-quality amplicons (i.e., did not pass the PacBio quality control), a total of 214 (88.4%) *ply*NCR amplicon products from 242 NS samples were sequenced (Fig. 3). This included PCR products from two of fourteen NS samples suspected of being VGS, due to having < 10 *lytA* copies and amplifying a short fragment with the wrong size (~ 1100 bp) during *ply*NCR PCR (Fig. 3). These two products were included to confirm the genomic signature differences between true *ply*NCR sequences and homolog sequences. Sequencing failed for only one of the 214 (0.5%) amplicons, despite using three replicates. Additional file 1: Table S6 summarizes the sequencing statistics and DADA2 denoising process for each SMRTcell.

### Sensitivity and resolution limit of PacBio sequenced *ply*NCR in mock control mixtures

**Fig. 4:**
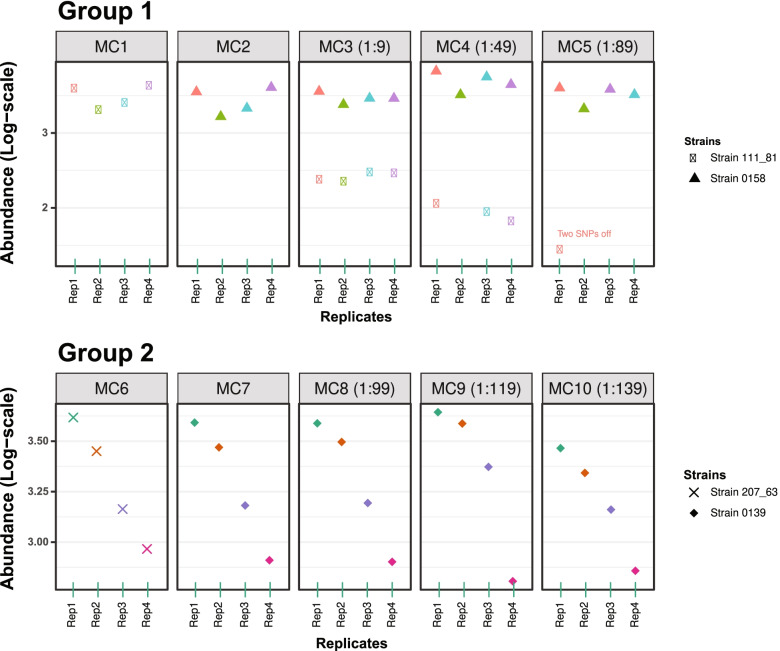
Sensitivity of the *ply*NCR for pneumococcal detection in sequenced mock samples. The *ply*NCR was amplified from 10 pneumococcal mock communities (MC) and sequenced in technical quadruplicates by SMRT sequencing. The top and bottom panels show the abundance of all strains recovered as amplicon sequence variants (ASVs) by DADA2 in MC1-5 (group 1) and MC6-10 (group 2), respectively. MC1, 2, 6, and 7 represent single strain communities. MC3, 4, and 5 contain mixtures of MC1 and 2 in ratios 1:9, 1:49, and 1:89, respectively, while MC8, 9, and 10 contain mixtures of MC6 and MC7 in ratios 1:99, 1:119, and 1:139, respectively. “Two SNPs off” indicates that the least abundant strain in MC5 (1:89) was recovered with all 17 expected SNPs and two additional, erroneous SNPs

To assess the sensitivity and resolution limit of the *ply*NCR for strain discrimination in a 25-sample-multiplex library, 10 mock community mixtures were sequenced in quadruplicates with NS amplicons, and strains from each community were resolved as amplicon sequence variants (ASVs) using DADA2. Figure 4 illustrates the results of the DADA2 error model. All strains were correctly identified down to the 2% variant level (1:49 mixture). The least abundant strain at the 1.1% variant level (1:89 mixture) was resolved in only one of four replicates, though with two additional, incorrect SNPs.

### The *ply*NCR marker enables pneumococcal strain-level identification in NS samples

**Fig. 5:**
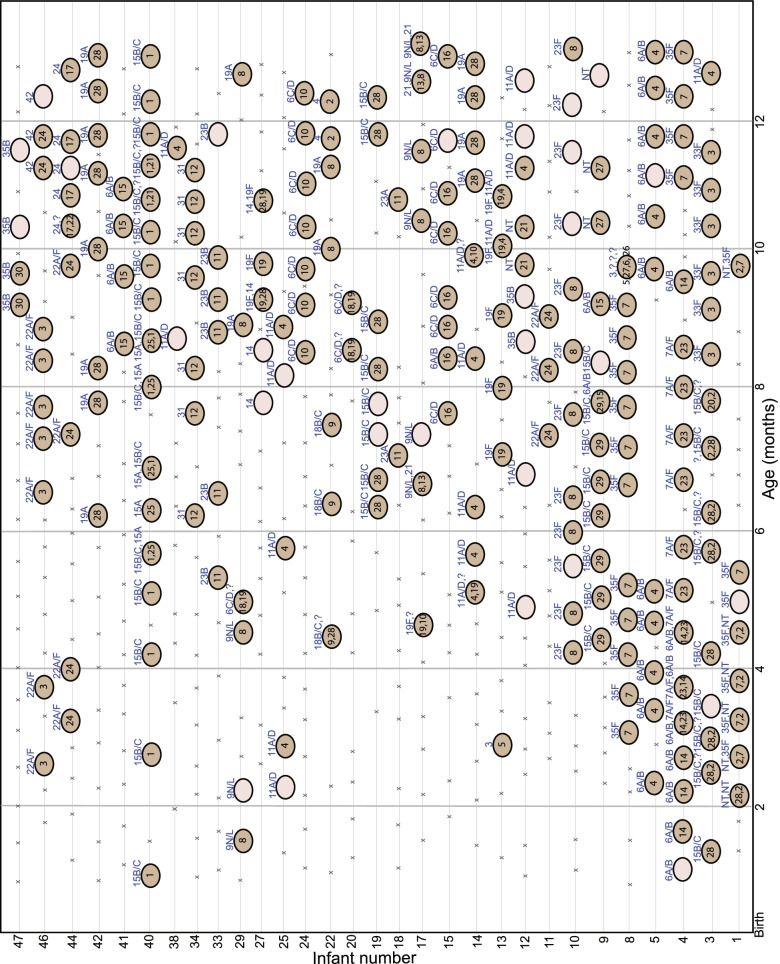
Non-culture-based Amplicon sequencing of the *ply*NCR marker for pneumococcal strain resolution. Nasal swabs (NS) are illustrated as crosses (“x”; *lytA* < 10 copies) or circles (*lytA* ≥ 10 copies) for pneumococcal absence and presence during the first year of life, respectively. Infants with only negative pneumococcal swabs were omitted. Each unique amplicon sequence variant (ASV) recovered by DADA2 after *ply*NCR sequencing represents a pneumococcal strain and is denoted as a single count (1–30) within the dark brown circles. The major (most abundant) *ply*NCR ASV is shown first, followed by minor (lower abundant) *ply*NCR ASVs. Swabs where pneumococci were expected (*lytA* ≥ 10 copies), but having no *ply*NCR ASV (due to failed/sub-optimal amplification, low QC quality, failed sequencing, or no DADA2 output) are illustrated as empty tan circles. Serotypes/serogroups identified via conventional multiplex PCR (cPCR) are shown at the top of each circle. Question mark (?) indicates an expected, but indeterminable serotype or non-typeable (NT) *S. pneumoniae* in a co-colonizing sample, due to the limitations of the multiplex panel

Given a demonstrated discriminatory power of the *ply*NCR in mock samples, sequenced *ply*NCR amplicons from NS samples were analyzed. In total, DADA2 identified 250 *ply*NCR ASVs from 209/213 (98%) sequenced samples. Thirty of these 250 (12%) ASVs were unique and represented distinct strain profiles in each infant during the 12-month period (Fig. 5). Co-colonization was detected in 38/213 (17.8%) sequenced samples with the relative proportion of the least abundant strain(s) ranging from 1.1 to 48.8% (median, 17.2%; IQR, 5.8–33.4%). No ASV output was observed in four samples; two of which (having < 10 *lytA* copies) had amplified a *ply*NCR homolog. On relaxation of the DADA2 read length stringency settings, all four samples outputted homolog sequences with an average read length of 1055 bp (range, 953–1072 bp), sharing high BLAST identities (range, 92.8–97%) to VGS species. This confirmed the two suspicious samples that amplified a *ply*NCR homolog to be indeed VGS species.

### Serotype identification of *ply*NCR ASVs using conventional PCR (cPCR)

Of the 240 NS samples having ≥ 10 *lytA* copies, a total of 24 serotypes/serogroups were identified via multiplex/singleplex cPCR. Overall, 59.3% (166/280) of serotypes were non-PCV13, 29.6% (83/280) were PCV13, 6.8% (19/280) were unknown, and 4.3% (12/280) were NT (Fig. 5; Additional file 1: Table S7). A distinct serotype or NT was linked to each unique *ply*NCR ASV strain profile in an infant, except for infants #15 and #29, where two distinct serotypes/serogroups were identified at different time points for their respective *ply*NCR ASV (#16 and #8) strain profiles, indicating new strain acquisitions (Fig. 5). New strain acquisitions were also inferred for infant #3, where the same serotype/serogroup was identified for two unique *ply*NCR ASVs (#28 and #20) (Fig. 5). Multiple serotype identification by cPCR was limited for 44.7% (17/38) of samples with identified co-colonization, preventing serotype attribution for 12 *ply*NCR ASVs in nine infants (Fig. 5; Additional file 1: Table S7). In addition, it is not possible to link the amplicon sequencing variants and serotypes in samples containing co-colonizing serotypes unless the major types are clearly more abundant as compared to the minor types.

### Pneumococcal carriage prevalence, acquisition, and dynamics

**Fig. 6:**
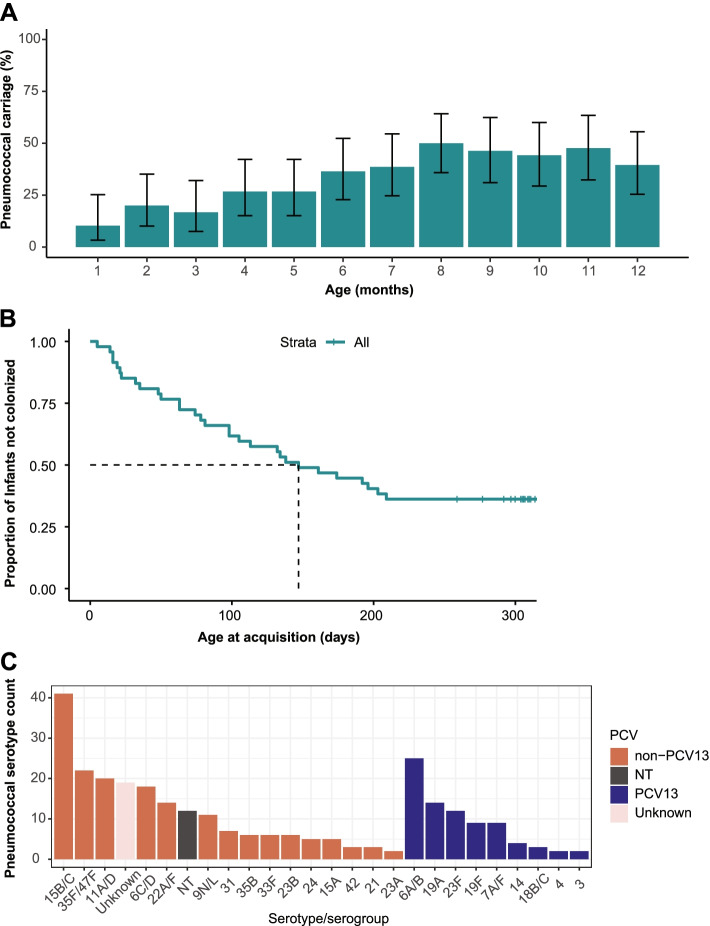
Pneumococcal carriage prevalence, acquisition, and dynamics. **A** Carriage prevalence by age. **B** Age at first acquisition for infants (dotted line shows median). **C** Serotype distribution

Overall, 30/47 (63.8%) infants carried pneumococci (*lytA*-based carriage) at least once during the 12-month study period. Carriage increased from 10.3% (4/39; 95% CI 3.3–25.2%) during the first month of enrollment (before 2 months of age), reaching a peak of 50% (22/44; 35.8–64.2%) during the 8-month of life (Fig. 6A).

The median age at first acquisition was 147 days (IQR 63–303 days) (Fig. 6B). Of the assessed cohort characteristics, having two or more siblings was found to be significantly associated with an increased rate of first acquisition (*p* = 0.02; Additional file 1: Table S8). Among those with carriage episodes, the number of observed pneumococcal acquisitions ranged from one to six per child, with a total of 86 pneumococcal acquisitions for all 47 infants.

The median duration of carriage for the first acquired pneumococcal episode was 98 days (IQR 28–140 days), compared to 41 days (25–75 days) for successive acquisitions (*p* = 0.054, log-rank test). Among serotypes, the median duration of carriage was longer for non-PCV13 serotypes at 71 days (IQR 41–101 days), compared to 63 days (31–148 days) for PCV13 serotypes (Additional file 1: Table S9). Serotype 15B/C was the most prevalent serotype among all infants (Fig. 6C). Co-colonization was observed at least once among 50% of infants carrying pneumococci. Majority (52.6%; 20/38) of strains exhibited a stable co-colonization dynamic, followed by strain dominance (28.9%), short-term co-colonization (13.2%), and displacement (5.3%) (Additional file 1: Fig. S3).

## Discussion

For the first time, we report an amplicon-based high-throughput sequencing (HTS) approach that incorporates the DADA2 algorithm, enabling pneumococcal strain resolution and longitudinal characterization of carriage dynamics based on SNPs in the *ply*NCR marker. Our method is less labor-intensive and more accurate than conventional methods, as it allows for direct detection of major and minor (low abundance) pneumococcal strain variants and, therefore, circumvents the need of picking and analyzing individual pneumococcal colonies to fully grasp the extent of diversity. Also, our method does not require prior culture enrichment which may disrupt the original bacterial composition as certain variants may outcompete others.

We first performed qPCR of *lytA* which is a gold standard method for the detection of *S. pneumoniae*. While it is known that the specificity is not 100%, it is very well accepted to use *lytA* for the detection of *S. pneumoniae* [22]. Our results further demonstrate the *ply*NCR’s potential as a top-performing pneumococcal marker with an in silico specificity among only *S. pneumoniae* strains as well as a high-molecular (PCR) performance (100% PPV and 100% specificity) matching that of the gold standard (*lytA* qPCR). The modest rates achieved in sensitivity (94.2%) and NPV (97.8%) appear to stem from the use of a conserved amount of template DNA (10 μl), which in light of the assay’s previously described detection limit (1 pg) at a higher DNA amount (31.4 μl) [13], may explain the reduced sensitivity and NPV. Nevertheless, compared to previous marker discoveries like the SPN9802 [28, 29] and SP2020 [30], the *ply*NCR shows more promise, given an absence of misidentified strains (false positives) during the molecular assessment, as well as its ability to easily discriminate pneumococci from closely related VGS, which may harbor *ply*NCR homologs at a size difference of ~ 200 bp. To our knowledge, this is the first carriage study that provides adequate resolution of “unique” nasopharyngeal pneumococcal strains in a longitudinal infant sampling without a prior culture step. Only two other studies have attempted to characterize *S. pneumoniae* at the strain level using infant longitudinal samples [3, 4]. However, both studies employed a metagenomic approach, which requires (i) a general enrichment process for *Streptococcus* sp., (ii) a high coverage that is mostly outbalanced by host DNA, (iii) a short-read sequencing library requiring assembly, and (iv) a more computationally intensive (and mostly database-dependent) approach [31], all of which may be cost-limiting for large-scale studies. On the other hand, our amplicon sequencing strategy utilizes a variable, species-specific marker that can be (i) amplified directly from clinical samples, (ii) sequenced as long reads on a 3rd generation platform (PacBio) with an accuracy of ≥ 99.8%, and (iii) easily resolved as true genetic strain variants down to a single nucleotide difference via the DADA2 pipeline. However, and as shown very recently, deep shotgun sequencing has of course unsurpassed sensitivity for detecting multiple colonization presupposed prior culturing maintains the original “in vivo” co-colonization ratio [32].

The parallel use of cPCR enabled serotype identification directly from NS samples and allowed serotypes to be primarily linked to most strain (*ply*NCR ASV) profiles. However, co-colonizing serotypes were unidentified in 45% of NS samples, demonstrating a significant limitation of this method for multiple serotype detection, as previously shown [2, 33]. In addition, it is not possible to unambiguously link the amplicon sequencing variants and serotypes in samples containing multiple samples. The microarray, which is currently considered the best method for detecting multiple serotypes [2], notably overcomes these limitations, as it is able to detect all known serotypes, including NTs and other bacterial species as well as their relative abundances, though at a higher cost and with a culture-amplification step, which is not required for cPCR. The same is true for the abovementioned very recent study using shotgun sequencing [32].

By itself, the *ply*NCR does not offer any serotype information for resolved strains in carriage, and likewise, serotyping does not provide any genomic background details for pneumococci in carriage. The advantage of the *ply*NCR for carriage studies is therefore complemented by serotyping, as it confirms the identity of a particular strain (*ply*NCR ASV) and allows instances of new acquisitions (with an identical serotype, but a different strain) and re-acquisitions to be easily identified. Thus, *ply*NCR combined with serotyping offers a more detailed insight into the carriage dynamics including duration.

Based on our findings, the median age to first acquisition (147 days) was much higher in our study compared to reports from higher carriage countries such as South Africa, Malawi, Indonesia, and the Gambia [25, 27, 34, 35]. Given higher rates of PCV uptake in Switzerland [36], the longer time to acquisition in our birth cohort may therefore reflect reduced chances for pneumococcal transmission. Also, the very first swab was taken once the infants were 6 weeks of age in our study. We may have missed earlier pneumococcal carriage, though pneumococcal colonization might be quite rare in the first days/weeks of life.

The presence of two or more siblings in the home was the only risk factor associated with the first pneumococcal acquisition, supporting previous reports of older siblings being the transmission sources for infants [37, 38]. We additionally observed a trend of longer carriage durations for the first acquired pneumococcal episode compared to subsequent episodes, which is similar to a recent study in Indonesia [27]. Longer duration of carriage has been shown to be positively correlated with more prevalent serotypes and negatively associated with age [39], which may partially explain our findings, as 66% of first acquisitions were the more prevalent non-PCV13 serotypes.

Contrary to a previous study, which demonstrated stabilized co-colonization rates during the pre-vaccine (2004–2005) and PCV7 era (2006–2009) in Switzerland [14], our current findings indicate that the rate of co-colonization is ~ 2× higher since PCV13 implementation (2010–2014) in the country. Interestingly, our estimated rate of 17.8% is not far off when compared to studies conducted during similar periods in the Netherlands (22%) [40], Portugal (25%) [41], and Nepal (20.2%) [42]. Although it remains unclear whether overall co-colonization rates are affected by PCV selection pressure or simply unmasking following the use of more sensitive detection measures, the lower rates observed in the previous Swiss study may imply an underestimation bias resulting from sampling acute otitis media (AOM) and pneumonia patients, who represent populations with compromised nasopharyngeal flora [43], compared to healthy (asymptomatic) young children, who are considered the major reservoir and drivers of pneumococcal transmission [38, 44]. At the same time, methodological differences may have also contributed to differing estimations.

The most common pneumococcal co-colonization dynamic observed in this birth cohort was a stable co-colonization, with varied relative strain abundances, indicating continued inter-strain competition. This is contrary to a recent study in Indonesian infants [27], where displacement of the primary colonizer by the secondary serotype was more common. As there is no national PCV program in Indonesia, a disparity in the serotype distributions of both countries may explain the observed differences in co-colonization dynamics. This is mostly evidenced by reported high carriage episodes of serotype 6B in Indonesia [27], which has all but disappeared from Swiss carriage isolates [45]. As the Indonesian study did not include any information on the serotypes involved in co-colonization, no further comparisons can be made between both studies. However, the identification of serotype 15B/C as a frequent initial/primary co-colonizer [41, 42] is consistent with the observations in this study.

Our study has a number of limitations. First, the NS samples used here were collected by the parents up to a decade ago, and as a result, we cannot rule out the possibility of a substandard collection as well as potential sample degradation from previous frequent use of the samples in earlier studies. We have chosen “deep nasal” over nasopharyngeal swabs due to logistical reasons as we told the parents rather than study nurses to weekly swab their children during the first year of life. However, we received a high pneumococcal carriage rate (~ 64%). In comparison, the pneumococcal carriage rate found by PCR was 51.6% in Swiss children in 2009, and we are therefore confident that our data does not underestimate the carriage rate [13]. Second, it is possible that the unavoidable addition of the PacBio Universal sequencing overhangs to the *ply*NCR primers may have reduced the amplification sensitivity, especially in the presence of low template DNA. This is mostly supported by successful PCR amplifications (without overhangs) in 50% of the 14 NS samples that failed to amplify with the use of overhang primers (results not shown). This trade-off in specificity due to the overhangs suggests that additional PCR optimization may be required for studies seeking to apply PacBio SMRT amplicon sequencing to their research. Third, there was no *ply*NCR ASV output for two samples, despite successful *ply*NCR amplification, suggesting a preferential sequencing bias for homologs, which were observed when the DADA2 length filter was lifted (results not shown). We are yet to estimate the frequency of this event in future studies. Fourth, sequencing had to be repeated for one amplicon, but no technical reason was found for its failure during troubleshooting. Therefore, the cost of PacBio amplicon sequencing could be exacerbated by random repetitions as well as the required sequencing depth for variant detection during multiplexing. However, this limitation is applicable to all HTS approaches. Lastly, a major limitation of this study was the absence of a culture-based serotyping method like the Quellung or microarray for cPCR verification. This was not feasible as the nasal swabs used in this study were not intended for the investigation of viable bacterial cultures. Thus, our use of the cPCR was therefore unavoidable, though we argue that despite its limitations, cPCR still provided reliable serotyping results due to our study’s close sampling time frame (biweekly swabs), where previous and/or ensuing time points with identical serotypes and *ply*NCR ASVs provided additional support and justification for serotype attribution, even in 84% of co-colonizing samples.

Despite these limitations, our amplicon-based sequencing approach was successful in resolving strain profiles of *S. pneumoniae* in the nasopharynx and distinguishing them from those of the viridans group streptococci. In addition, our infant cohort represents an intensively sampled study population, preventing underestimation bias in carriage estimates and allowing us to closely track changing carriage and co-colonization dynamics over a 12-month period.

## Conclusions

We therefore conclude that the use of the *ply*NCR as an amplicon marker can further our understanding of carriage and co-colonization dynamics on a strain level. In so doing, we demonstrated pneumococcal strain acquisitions as unique *ply*NCR sequences with associated serotypes in an infant birth cohort and presented accurate estimations for carriage acquisition and durations on a strain level. As PCV13 implementation had and still has an impact on pneumococcal carriage (and therefore invasive diseases), consistent monitoring and increased follow-up remain a priority for understanding the carriage strain dynamics in a future era of next-generation vaccines.

## Supplementary Information

**Additional file 1: Table S1.** Details of the in-house pneumococcal strains used for mock community preparation. **Table S2.** Pacbio SMRTcell Sample pooling. **Table S3.** Assembly details of S. mitis, S. pseudopneumoniae and S. pneumoniae strains showing close plyNCR relatedness during in-silico analysis. **Table S4.** Characteristics of the BILD infant cohort (n=47). **Table S5.** Comparison of discordant qPCR and PCR amplification results of nasal swab samples (n=25) from BILD cohort infants. **Table S6.** Mean read output statistics from PacBio SMRT sequencing and DADA2 pipeline. **Table S7.** Conventional serotyping of nasal swab (NS) samples (n=240) from BILD cohort infants. **Table S8.** Multivariable Cox regression analysis of potential risk factors associated with time to first pneumococcal acquisition. A Hazard Ratio (HR) < 1 indicates a reduced hazard (rate) of pneumococcal acquisition while an HR > 1 indicates an increased rate of pneumococcal acquisition. **Table S9.** Duration of pneumococcal carriage by acquisition and serotype. **Fig. S1.** False positive removal during DADA2 ASV calling. **Fig. S2.** Detection of S. pneumoniae by plyNCR PCR. **Fig. S3.** Patterns of co-colonization dynamics in four infants.

## Data Availability

All raw PacBio sequencing reads were deposited in the European Nucleotide Archive (ENA) under study accession number PRJEB45241.
