# Supplementary material for: Strain-level resolution and pneumococcal carriage dynamics by single-molecule real-time (SMRT) sequencing of the plyNCR marker: a longitudinal study in Swiss infants
Source: Microbiome. 2022 Sep 22;10:152. doi: 10.1186/s40168-022-01344-6 (PMC9502908; doi:10.1186/s40168-022-01344-6)

**Supplementary Tables**

| Mock community (MC) | Strain ID | Sample_accession | Run_accession | Assembly ID | SNP difference | Isolation year | Isolation site | MLST | Country |
| --- | --- | --- | --- | --- | --- | --- | --- | --- | --- |
| MC1 | 111_81 | SAMEA1022851 | ERR097266 | 5737_3_10 | 17 | 1999 | Nasopharynx | 344 | Switzerland |
| MC2 | 0158 | SAMEA1022909 | ERR097291 | 5737_7_2 |  | 2008 | Nasopharynx | 4390 | Thailand |
| MC6 | 207_63 | SAMEA1022896 | ERR097261 | 5737_3_5 | 14 | 2001 | Nasopharynx | 344 | Switzerland |
| MC7 | 0139 | SAMEA1022780 | ERR097360 | 5919_1_11 |  | 2007 | Nasopharynx | 4130 | Thailand |

**Table S1.** **Details of the in-house pneumococcal strains used for mock community preparation**

**Table S2. Pacbio SMRTcell Sample pooling**

| **SMRTcell** | **Samples on SMRTcell** |
| --- | --- |
| SMRTcell 1 | 20 Samples + MC1-5 |
| SMRTcell 2 | 20 Samples + MC1-5 |
| SMRTcell 3 | 20 Samples + MC1-5 |
| SMRTcell 4 | 20 Samples + MC1-5 |
| SMRTcell 5 | 20 Samples + MC6-10 |
| SMRTcell 6 | 20 Samples + MC6-10 |
| SMRTcell 7 | 20 Samples + MC6-10 |
| SMRTcell 8 | 20 Samples + MC6-10 |
| SMRTcell 9 | 24 Samples |
| SMRTcell 10 | 26 Samples |
| SMRTcell 11* | 25 Samples (only 5 are *ply*NCR) |
| *20 samples in this SMRTcell did not belong to this study. The terms "sample" and "amplicon" are used interchangeably here. | |

**Table S3. Assembly details of S. mitis, S. pseudopneumoniae and S. pneumoniae strains showing close plyNCR relatedness during in-silico analysis.**

| **Strain ID** | **Assembly Accession** | **Organism** |
| --- | --- | --- |
| G42 | GCA_000506665.1 | *S. pseudopneumoniae* |
| 5247 | GCA_000506745.1 | *S. pseudopneumoniae* |
| 163_SPSE | GCA_001070715.1 | *S. pseudopneumoniae* |
| SMRU2944 | GCA_001115245.1 | *S. pseudopneumoniae* |
| SMRU2248 | GCA_001121785.1 | *S. pseudopneumoniae* |
| SMRU689 | GCA_001193755.1 | *S. pseudopneumoniae* |
| Type strain: N | GCA_001147205.1 | *S. pseudopneumoniae* |
| SMRU688 | GCA_001206055.1 | *S. pseudopneumoniae* |
| SMRU856 | GCA_001337855.1 | *S. pseudopneumoniae* |
| SMRU737 | GCA_001339755.1 | *S. pseudopneumoniae* |
| Spain939 | GCA_003598195.1 | *S. pseudopneumoniae* |
| CCUG 50868 | GCA_011714915.1 | *S. pseudopneumoniae* |
| SK597 | GCA_000148545.2 | *S. mitis* |
| DD28 | GCA_001579045.1 | *S. mitis* |
| SK564 | GCA_000148525.2 | *S. mitis* |
| NCTC11189 | GCA_901542415.1 | *S. mitis* |
| SK1080 | GCA_000220085.2 | *S. mitis* |
| Nm-65 | GCA_014467095.1 | *S. mitis* |
| SMRU824 | GCF_001096185.1 | *S. pneumoniae* |
| SMRU2014 | GCF_001113365.1 | *S. pneumoniae* |
| 0238 | GCF_001142105.1 | *S. pneumoniae* |
| SMRU1235 | GCF_001154565.1 | *S. pneumoniae* |
| SMRU1537 | GCF_001155165.1 | *S. pneumoniae* |

**Table S4. Characteristics of the BILD infant cohort (n=47)**

| **Characteristic** | **Summary (N=47)** |
| --- | --- |
| Female sex, n (%) | 23 (49) |
| C-section, n (%) | 8 (17) |
| **Season of birth, n (%)** |  |
| Spring | 12 (25.5) |
| Summer | 13 (27.7) |
| Fall | 11 (23.4) |
| Winter | 11 (23.4) |
| ^a^Smoking exposure in first year, n (%) | 7 (14.9) |
| ^b^Maternal atopy, n (%) | 12 (25.5) |
| ^c^Childcare, n (%) | 11 (23.4) |
| ^d^**Parental education, n (%)** |  |
| Low | 6 (12.8) |
| Middle | 18 (38.3) |
| High | 23 (48.9) |
| **Siblings, n (%)** |  |
| 0 | 10 (21.3) |
| 1 | 24 (51.1) |
| ≥2 | 13 (27.7) |
| Nasal swab sampling (high-quality), mean (SD) | 19 (±3.2) |
| Gestational age at birth [weeks], mean (±SD) | 39.7 (±1.6) |
| Weight at birth [g], mean (±SD) | 3400 (±478) |
| Length at birth [cm], mean (±SD) | 49.7 (±2) |
| Breastfeeding duration [months], mean (±SD) | 8.9 (±2.7) |
| **^‡^Age at PCV administration [weeks], mean (±SD)** |  |
| First dose | 10.2 (±3.6) |
| Second dose | 19 (±4.1) |
| **Respiratory symptoms, n, mean (±SD)** |  |
| Rhinitis | 260, 5.5 (±2.5) |
| ^e^URTI | 168, 3.6 (±2.3) |
| ^f^LRTI | 31, 0.7 (±1.1) |
| Wheezing | 10, 0.2 (±0.6) |
| SD: Standard deviation.  ^a^Smoking exposure to father/mother during the first year of life.  ^b^Maternal atopy defined as asthma, hay fever or eczema.  ^c^Childcare at any timepoint during the first year of life.  ^d^Parent's education level was categorized as low (<4 years of apprenticeship), middle (≥4 years of apprenticeship) or high (tertiary education)  ^e^URTI: Upper respiratory tract infection symptoms were defined as coughing and/or wheezing.  ^f^LRTI: Lower respiratory tract infection symptoms were defined as presence of cough, wheeze or breathing difficulties with URTI or fever for more than two consecutive days.  ‡ The pneumococcal conjugate vaccine (PCV) follows a 2+1 dosing schedule in Switzerland conducted at 2, 4 and 12 months of age. No study infant received the third (booster) PCV dose before 12 months of age. Five infants did not receive any PCV dose. One infant only received the first dose. | |

**Table S5. Comparison of discordant qPCR and PCR amplification results of nasal swab samples (n=25) from BILD cohort infants**

|  |  | ***lytA* qPCR**  **(≥10 copies)*** | **PCR** | |
| --- | --- | --- | --- | --- |
| **ID** | **Age (weeks)^a^** |  | ***ply*NCR^b^** | ***cpsA**** |
| Infant1 | 19.7 | + | - | + |
| Infant3 | 14.1 | + | FA | + |
| Infant4 | 4.7 | + | - | + |
| Infant9 | 33.3 | + | FA | + |
| Infant10 | 45.6 | + | FA | + |
| Infant12 | 19.9 | + | - | + |
| Infant12 | 29 | + | - | + |
| Infant12 | 35 | + | - | + |
| Infant12 | 37.1 | + | - | + |
| Infant12 | 47 | + | - | + |
| Infant12 | 51 | + | - | + |
| Infant15 | 46.7 | + | - | + |
| Infant17 | 30.1 | + | FA | + |
| Infant19 | 29.3 | + | FA | + |
| Infant19 | 31.3 | + | - | + |
| Infant25 | 9.4 | + | FA | + |
| Infant25 | 32.1 | + | - | + |
| Infant27 | 31.4 | + | FA | + |
| Infant27 | 33.4 | + | FA | + |
| Infant29 | 8.4 | + | FA | + |
| Infant33 | 46.9 | + | - | + |
| Infant38 | 35.7 | + | - | + |
| Infant44 | 45.6 | + | FA | + |
| Infant46 | 48.9 | + | FA | + |
| Infant47 | 46.3 | + | - | + |
| ^a^Age at timepoint of sample collection. ^b,^ * FA, plus and negative signs indicate faint, positive and negative amplification, respectively. | | | | |

**Table S6.** **Mean read output statistics from PacBio SMRT sequencing and DADA2 pipeline**

|  | | | | **DADA2 pipeline** | | |
| --- | --- | --- | --- | --- | --- | --- |
| **SMRTcell** | **Subreads** | **Aligned** | **CCS^1^** | **Primer removal** | **Filtered** | **Denoised** |
| SMRTcell1 | 631150 ± 208378 | 586880 ± 195158 | 6862 ± 2291 | 5929 ± 1956 | 4299 ± 1414 | 4295 ± 1414 |
| SMRTcell2 | 505302 ± 183076 | 482087 ± 174850 | 4257 ± 1557 | 3669 ± 1319 | 2603 ± 946 | 2596 ± 945 |
| SMRTcell3 | 673396 ± 231203 | 641277 ± 218518 | 6164 ± 2088 | 5387 ± 1811 | 3790 ± 1266 | 3783 ± 1261 |
| SMRTcell4 | 454849 ± 218766 | 420939 ± 196120 | 4859 ± 2146 | 4201 ± 1876 | 3021 ± 1333 | 3001 ± 1327 |
| SMRTcell5 | 323832 ± 152082 | 304359 ± 151478 | 3169 ± 1629 | 2764 ± 1510 | 1974 ± 1072 | 1969 ± 1070 |
| SMRTcell6 | 431725 ± 252922 | 402173 ± 244528 | 4565 ± 2659 | 3908 ± 2234 | 2790 ± 1592 | 2786 ± 1591 |
| SMRTcell7 | 416651 ± 250590 | 385574 ± 235306 | 4191 ± 2486 | 3480 ± 2022 | 2474 ± 1435 | 2469 ± 1429 |
| SMRTcell8 | 440300 ± 284237 | 422858 ± 273229 | 2740 ± 1702 | 2267 ± 1379 | 1472 ± 895 | 1470 ± 894 |
| SMRTcell9 | 515926 ± 256945 | 503411 ± 253901 | 5227 ± 2501 | 4696 ± 2256 | 3327 ± 1589 | 3299 ± 1589 |
| SMRTcell10 | 641340 ± 207674 | 624015 ± 203897 | 6559 ± 2102 | 5984 ± 1913 | 4266 ± 1365 | 4249 ± 1357 |
| SMRTcell11* | 411038 ± 433905 | 311370 ± 344933 | 3040 ± 3631 | 2650 ± 3184 | 2095 ± 2552 | 2079 ± 2541 |
| Average read output in a 25-26 pooled SMRTcell expressed as ± Standard Deviation (SD). ^1^ Circular Consensus Sequence  *Estimation for SMRTcell includes 20 samples unrelated to this study. | | | | | | |

**Table S7. Conventional serotyping of nasal swab (NS) samples (n=240) from BILD cohort infants.**

| **Infant ID** | ***ply*NCR ASV^1^** | **# of NS samples** | ***ply*NCR ASV carriage type^2^** | | **Serotype/Serogroup^3^** |
| --- | --- | --- | --- | --- | --- |
|  |  |  | **Single** | **Co-colonization** |  |
| #1 | 28 | 1 | - | + | NT |
|  | 2 | 6 | - | + | NT |
|  | 7 | 6 | + | + | 35F/47F |
|  | 0 | 1 | N/A | N/A | 35F/47F |
| #3 | 28 | 7 | + | + | 15B/C |
|  | 2 | 6 | - | + | ? |
|  | 0 | 1 | N/A | N/A | 15B/C |
|  | 20 | 1 | - | + | 15B/C |
|  | 3 | 6 | + | - | 33F |
|  | 4 | 1 | + | - | 11A/D |
| #4 | 0 | 1 | N/A | N/A | 6A/B |
|  | 14 | 7 | + | + | 6A/B |
|  | 23 | 9 | + | + | 7A/F |
|  | 7 | 4 | + | - | 35F/47F |
| #5 | 4 | 10 | + | - | 6A/B |
|  | 0 | 1 | N/A | N/A | 6A/B |
| #8 | 7 | 11 | + | - | 35F/47F |
|  | 5 | 1 | - | + | 3 |
|  | 27 | 1 | - | + | ? |
|  | 6 | 1 | - | + | ? |
|  | 26 | 1 | - | + | ? |
| #9 | 29 | 7 | + | + | 15B/C |
|  | 15 | 2 | + | + | 6A/B |
|  | 0 | 1 | N/A | N/A | 15B/C |
|  | 27 | 2 | + | - | NT |
|  | 0 | 1 | N/A | N/A | NT |
| #10 | 8 | 8 | + | - | 23F |
|  | 0* | 2 | N/A | N/A | 23F |
|  | 0 | 2 | N/A | N/A | 23F |
| #11 | 24 | 3 | + | - | 22A/F |
| #12 | 0 | 4 | N/A | N/A | 11A/D |
|  | 0 | 2 | N/A | N/A | 35B |
|  | 21 | 2 | + | - | NT |
|  | 4 | 1 | + | - | 11A/D |
| #13 | 5 | 1 | + | - | 3 |
|  | 19 | 5 | + | + | 19F |
|  | 4 | 2 | - | + | 11A/D |
| #14 | 4 | 5 | + | + | 11A/D |
|  | 19 | 1 | - | + | ? |
|  | 10 | 1 | - | + | ? |
|  | 28 | 4 | + | - | 19A |
| #15 | 16 | 7 | + | - | 6C/D (6), 6A/B (1) |
|  | 0 | 1 | N/A | N/A | 6C/D |
| #17 | 19 | 1 | - | + | 19F |
|  | 10 | 1 | - | + | ? |
|  | 8 | 5 | + | + | 9N/L |
|  | 13 | 3 | - | + | 21 |
|  | 0 | 1 | N/A | N/A | 9N/L |
| #18 | 11 | 2 | + | - | 23A |
| #19 | 28 | 6 | + | - | 15B/C |
|  | 0 | 2 | N/A | N/A | 15B/C |
| #20 | 18 | 2 | - | + | 6C/D |
|  | 19 | 2 | - | + | ? |
| #22 | 9 | 3 | + | + | 18B/C |
|  | 28 | 1 | - | + | ? |
|  | 8 | 2 | + | - | 19A |
|  | 2 | 2 | + | - | 4 |
| #24 | 10 | 7 | + | - | 6C/D |
| #25 | 0 | 2 | N/A | N/A | 11A/D |
|  | 4 | 3 | + | - | 11A/D |
| #27 | 0 | 2 | N/A | N/A | 14 |
|  | 19 | 3 | + | + | 19F |
|  | 28 | 2 | - | + | 14 |
| #29 | 8 | 4 | + | - | 9N/L (2), 19A (2) |
|  | 0 | 1 | N/A | N/A | 9N/L |
|  | 18 | 1 | - | + | 6C/D |
|  | 19 | 1 | - | + | ? |
| #33 | 11 | 5 | + | - | 23B |
|  | 0 | 1 | N/A | N/A | 23B |
| #34 | 12 | 7 | + | + | 31 |
| #38 | 4 | 1 | + | - | 11A/D |
|  | 0 | 1 | N/A | N/A | 11A/D |
| #40 | 1 | 16 | + | + | 15B/C |
|  | 25 | 5 | + | + | 15A |
|  | 21 | 2 | - | + | ? |
| #41 | 15 | 4 | + | - | 6A/B |
| #42 | 28 | 8 | + | - | 19A |
| #44 | 24 | 4 | + | - | 22A/F |
|  | 17 | 4 | + | + | 24 |
|  | 22 | 1 | - | + | ? |
|  | 0 | 1 | N/A | N/A | 24 |
| #46 | 3 | 7 | + | - | 22A/F |
|  | 24 | 2 | + | - | 35A/C/42 |
|  | 0 | 1 | N/A | N/A | 35A/C/42 |
| #47 | 30 | 2 | + | - | 35B |
|  | 0 | 2 | N/A | N/A | 35B |
| ^1^ASV- Amplicon Sequence Variant outputs from DADA2. The 30 unique ASVs are numbered from 1-30. 0 indicates samples, where *ply*NCR sequencing was not possible (either due to failed/sub-optimal amplification or low QC quality*)*, thus no ASV. 0* indicates samples with successful *ply*NCR sequencing, but no *ply*NCR ASV output during DADA2.  ^2^Presence or absence of single or multiple (co-colonization) ASVs at one sample timepoint is denoted by positive (+) and negative (-) signs. The non-applicable (N/A) sign is used for samples with no ASVs.  ^3^Final serotype/serogroups were defined using conventional PCR (cPCR; multiplex/singleplex). The final serotype could not be determined for some minor (low abundance) ASVs found only during co-colonization (highlighted in yellow). Serotype and # of NS samples indicated in [] and (), respectively. | | | | | |

**Table S8: Multivariable Cox regression analysis of potential risk factors associated with time to first pneumococcal acquisition. A Hazard Ratio (HR) < 1 indicates a reduced hazard (rate) of pneumococcal acquisition while an HR > 1 indicates an increased rate of pneumococcal acquisition.**

| **Characteristic** | **Hazard ratio (95% CI)** | ***P* value** |
| --- | --- | --- |
| Pre-PCV13 era | 1.41 (0.54-3.64) | 0.48 |
| Rhinitis symptoms | 2.44 (0.80-7.4) | 0.12 |
| ^a^URTI symptoms | 0.35 (0.09-1.36) | 0.13 |
| Smoking exposure in home | 0.88 (0.25-3.16) | 0.85 |
| ≥2 siblings | 3.08 (1.19-7.93) | **0.02** |
| ^b^Parental education | 1.34 (0.36-4.96) | 0.66 |
| Fall/winter season of birth | 0.65 (0.26-1.60) | 0.35 |
| 2 dose PCV vaccination | 0.75 (0.26-2.18) | 0.59 |
| *P* < 0.05 in bold.  ^a^URTI: Upper respiratory tract infection symptoms were defined as coughing and/or wheezing.  ^b^Parent's education level was categorized as low (<4 years of apprenticeship), middle (≥4 years of apprenticeship) or high (tertiary education) | | |

**Table S9. Duration of pneumococcal carriage by acquisition and serotype**

|  | **Number of new acquisitions** | **Median carriage duration (days)** | **95% CI** |
| --- | --- | --- | --- |
| ***Acquisition*** | | | |
| 1^st^ | 30 | 98 | 56-146 |
| 2^nd^ | 20 | 63 | 51-121 |
| 3^rd^ | 15 | 39 | 28-N/A |
| 4^th^ | 9 | 32 | 21-N/A |
| 5^th^ | 6 | 60 | 14-N/A |
| 6^th^ | 1 | N/A | N/A |
| ***Serotype*** | | | |
| All | 81 | 59 | 41-87 |
| ^a^Non-PCV13 | 45 | 71 | 41-101 |
| ^b^PCV13 | 19 | 63 | 31-148 |
| ^c^Unknown (?) | 13 | 32 | 14-NA |
| Non-typeable (NT) | 4 | 59 | 12-NA |
| ^a^Serotypes not included in the PCV13 vaccine formulation.  ^b^Serotypes included in the PCV13 vaccine formulation.  ^c^Secondary (2^nd^/3^rd^/ 4^th^) serotypes could not be ascertained for some NS samples. | | | |

**Supplementary Figures**

**Figure S1. False positive removal during DADA2 ASV calling.**

**Pick top 2 ASVs**

**NO**

**YES**

**NO**

**YES**

**NO**

**DADA2**

**No co-colonization**

**False positive**

**True co-colonization**

**YES**

**Relative abundance of 2nd ASV ≥1%?**

**Figure S2. Detection of S. pneumoniae by plyNCR PCR**

*PCR-based detection of Streptococcus pneumoniae in nasal swab (NS) samples of infants*. Agarose gel electrophoresis of *ply*NCR PCR products are shown on a 1% gel. PCR products of *S. pneumoniae* (~1300 bp) are shown on lanes 1, 4 and 5. PCR product of viridans group streptococci (VGS; ~1100 bp) are shown on lanes 2 and 3. M: DNA ladder marker.


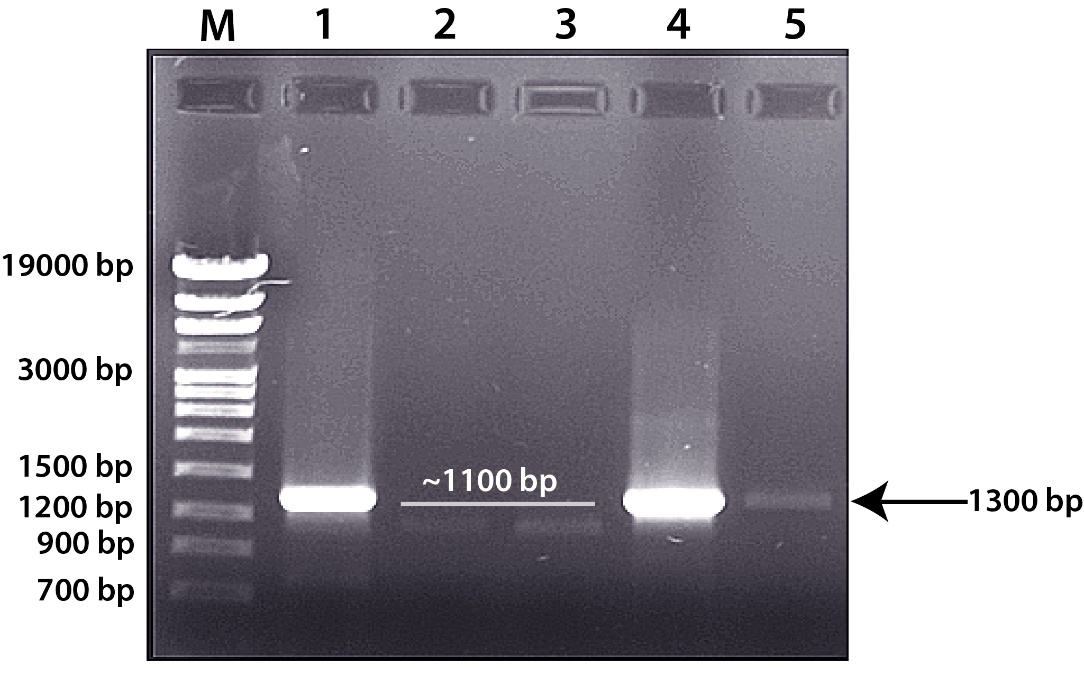


**Figure S*3*. Patterns of co-colonization dynamics in four infants.**

Due to a lack of confirmation in previous/ensuing timepoints, serotype 3 is assumed to be linked to the most abundant plyNCR ASV (ASV #5) for infant 8 in graph.


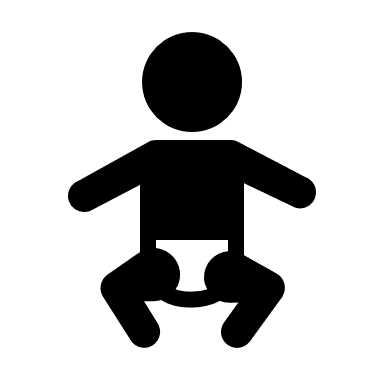

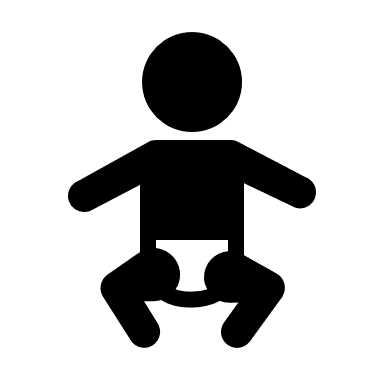

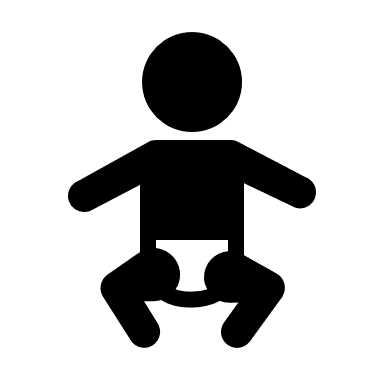

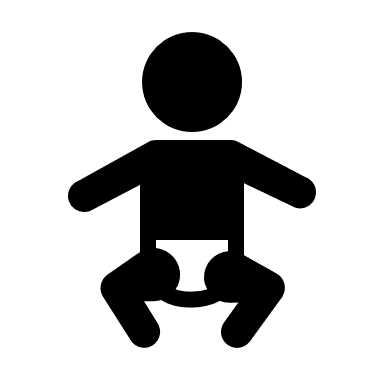

Supplement: Supplementary file 2 — Additional file 1: Table S1. Details of the in-house pneumococcal strains used for mock community preparation. Table S2. Pacbio SMRTcell Sample pooling. Table S3. Assembly details of S. mitis, S. pseudopneumoniae and S. pneumoniae strains showing close plyNCR relatedness during in-silico analysis. Table S4. Characteristics of the BILD infant cohort (n=47). Table S5. Comparison of discordant qPCR and PCR amplification results of nasal swab samples (n=25) from BILD cohort infants. Table S6. Mean read output statistics from PacBio SMRT sequencing and DADA2 pipeline. Table S7. Conventional serotyping of nasal swab (NS) samples (n=240) from BILD cohort infants. Table S8. Multivariable Cox regression analysis of potential risk factors associated with time to first pneumococcal acquisition. A Hazard Ratio (HR) < 1 indicates a reduced hazard (rate) of pneumococcal acquisition while an HR > 1 indicates an increased rate of pneumococcal acquisition. Table S9. Duration of pneumococcal carriage by acquisition and serotype. Fig. S1. False positive removal during DADA2 ASV calling. Fig. S2. Detection of S. pneumoniae by plyNCR PCR. Fig. S3. Patterns of co-colonization dynamics in four infants. [file 40168_2022_1344_MOESM1_ESM.docx]
